# Caries Experience Differs between Females and Males across Age Groups in Northern Appalachia

**DOI:** 10.1155/2015/938213

**Published:** 2015-05-27

**Authors:** John R. Shaffer, Elizabeth J. Leslie, Eleanor Feingold, Manika Govil, Daniel W. McNeil, Richard J. Crout, Robert J. Weyant, Mary L. Marazita

**Affiliations:** ^1^Department of Human Genetics, Graduate School of Public Health, University of Pittsburgh, Pittsburgh, PA 15261, USA; ^2^Center for Craniofacial and Dental Genetics, Department of Oral Biology, School of Dental Medicine, University of Pittsburgh, Pittsburgh, PA 15261, USA; ^3^Department of Biostatistics, Graduate School of Public Health, University of Pittsburgh, Pittsburgh, PA 15261, USA; ^4^Dental Practice and Rural Health, West Virginia University School of Dentistry, Department of Psychology, Eberly College of Arts and Sciences, West Virginia University, Morgantown, WV 26506, USA; ^5^Department of Periodontics, West Virginia University School of Dentistry, Morgantown, WV 26506, USA; ^6^Department of Dental Public Health and Information Management, School of Dental Medicine, University of Pittsburgh, Pittsburgh, PA 15261, USA; ^7^Clinical and Translational Science Institute and Department of Psychiatry, School of Medicine, University of Pittsburgh, Pittsburgh, PA 15261, USA

## Abstract

Sex disparities in dental caries have been observed across many populations, with females typically exhibiting higher prevalence and more affected teeth. In this study we assessed the sex disparities in two Northern Appalachian populations from West Virginia (WV, *N* = 1997) and Pennsylvania (PA, *N* = 1080) by comparing caries indices between males and females across four phases of dental development: primary dentition in children aged 1–5 years, mixed dentition in children aged 6–11 years, permanent dentition in adolescents aged 12–17 years, and permanent dentition in adults aged 18–59 years. No significant sex differences were observed for children aged 1–5 years. Contrary to national and international trends, WV girls aged 6–11 years had 1.5 fewer affected teeth than boys (*p* < 0.001). However, by ages 12–17, caries indices in the WV girls matched those in boys. In both WV and PA adults, women and men had similar total counts of affected teeth (i.e., DMFT), although women had more dental restorations (*p* < 0.001) and men had more current decay (*p* < 0.001). These results suggest that in some Appalachian populations, young girls benefit from protection against caries that is lost during adolescence and that adult women utilize dental health care to a greater degree than men.

## 1. Introduction

Dental caries (i.e., tooth decay) is the most common chronic disease worldwide, and one that exhibits profound disparities between affluent and impoverished nations and between privileged and disadvantaged populations within wealthy nations [1]. In the USA, for example, untreated dental caries and negative concomitants (i.e., pain, absenteeism from school or work, difficulty of chewing, sleep disturbance, poor self-image, poor social relationships, and tooth loss) disproportionately affect racial minorities and those living in poverty and rural communities. Sex differences in dental caries experience have also been widely observed, with most studies showing that women and girls are at higher risk and experience more carious lesions than do men and boys [2, 3].

The factors that cause women and girls to experience a greater burden of dental caries are not fully understood, and some of these factors may differ among populations. Possible explanations have been proposed, including earlier tooth eruption in girls (and therefore increased time of exposure to cariogenic processes), differences in dietary behaviors, access and utilization of oral health care, hormonal and/or physiological differences, and characteristics of the dentition, tooth enamel, or saliva [2, 3]. Others have proposed that the differential effects of genes influencing dental caries may partly explain the observed sex differences [4, 5]. We have previously demonstrated significant differences in genetic susceptibility to dental caries between the sexes using family based methods [6]; however, genetics only explains part of the differences in caries experience between males and females. Many questions remain, including which exogenous factors are most important, whether these differ among populations, and how these can be remedied to reduce sex disparities. Furthermore, the differences in dental caries experience between the sexes have yet to be characterized for some underserved populations.

To address this issue, we performed an assessment of sex differences in dental caries experience in the Center for Oral Health Research in Appalachia (COHRA), cohort 1 (COHRA1). The Appalachian region of the USA, which spans multiple states and includes urban foci among suburban and rural expanses, contains population groups with some of the poorest oral health indices in the nation [7–13]. In order to assess the potential disparities facing women and girls in the Northern Appalachian region that is the focus of COHRA, we compared males and females for untreated and treated dental decay across ages and across dentitions in two distinct populations from West Virginia (WV) and Pennsylvania (PA).

## 2. Methods

### 2.1. Participant Recruitment and Generalizability

COHRA was developed as a joint initiative between West Virginia University and the University of Pittsburgh to investigate the factors contributing to oral health disparities in Appalachia. Participants for the COHRA1 cohort were recruited from regions of two Northern Appalachian states with key demographic differences. The WV sample comprised participants from rural, predominantly non-Hispanic white communities from two representative counties (Webster and Nicholas) with low mean socioeconomic status and greater geographic barriers to oral health care. The PA sample comprised participants from three lower- to middle-class rural (Burgettstown and Bradford) and urban (Braddock) communities, which were also predominantly non-Hispanic white although Burgettstown and Braddock included substantial numbers of blacks as well as some participants self-reporting as “other” or not reporting any racial or ethnic affiliation. Reflecting the overall goal of COHRA to address individual-, family-, and community-level factors influencing oral health, participants in the COHRA1 cohort were enrolled according to a household-based strategy that targeted biological parent-child pairs, with all additional household members invited to participate regardless of biological or legal relationships. The full study protocol has been published previously [14]. All adult participants provided written informed consent and all children provided assent with parental or legal guardian informed consent. All study protocols were approved by the Institutional Review Boards at West Virginia University and the University of Pittsburgh.

Efforts were taken to accrue samples of volunteers that were as representative as possible of the respective Appalachian communities in WV and PA. In WV, this was achieved through coordination with two community partners: (1) the West Virginia University Prevention Research Center, which is a Center for Disease Control and Prevention- (CDC-) funded body devoted to expanding community engagement in health policy, practice, and research and (2) the West Virginia Rural Health Education Partnerships program, which was an initiative devoted to coordinating and expanding the network of health care practices reaching underserved communities in the state. Recruitment strategies were designed to reach potential participants across the spectrum of ages and geographic and socioeconomic categories of the target population. These efforts included flyers, print and radio advertisements, school-based presentation to teachers and parents of preschool, primary, secondary, and high school students, participation at health fairs, presentations to faith-based organizations, recruitment through WV Women, Infants, and Children Program clinics, WV Rural Health Education Partnership clinics, and other social service associations, and conferences.

These strategies yielded a sample that spanned ages and socioeconomic status groups and that was, on average, similar in regard to income (i.e., median annual household income <$25,000 in the sample) and educational attainment (i.e., majority high school graduate among adults) to US Census data for these areas. However, it is important to note that the COHRA1 study did not utilize statistical sampling, and therefore the COHRA1 cohort is not necessarily representative of the underlying population in all dimensions. Moreover, Appalachia is a vast geographic area comprising heterogeneous populations, and therefore inferences obtained in COHRA1 are not necessarily generalizable to other communities within the Appalachian region, nor the US population at large. Lastly, the COHRA1 study design did not seek capturing data on adults without children nor adults with adult-aged offspring.

### 2.2. Data Collection

Intraoral examinations by licensed dentists or research dental hygienists were conducted for each study participant. Presence or absence of each tooth was noted, and for each missing tooth the participant-reported cause (i.e., decay, trauma, periodontics-related extraction, periodontal disease, etc.) was noted. Each surface or each present tooth was examined for evidence of current decay (including precavitated “white spot” lesions) or restorations of past decay. Assessments excluded the third molars of the permanent dentition. Several dental caries indices were generated from these assessments. For the permanent dentition we considered the numbers of missing teeth due to decay (MT), restored filled teeth (FT), decayed teeth (DT) (excluding white spots), and decayed teeth including white spots (DWT). Similar indices were generated for the primary dentition: mt, ft, dt, and dwt. Likewise, traditional DMFT and dft indices were generated, as well as analogous indices that also included white spot lesions: DWMFT and dwft. Reproducibility of dental caries assessments was high, with inter- and intraexaminer concordances from 0.86 to 0.99 [14, 15].

### 2.3. Statistical Analysis

In this study, we investigated sex differences in mean caries indices separately in WV and PA samples, as well as three geographic subsamples within PA. Analyses were performed for four age ranges (rounding down to the nearest whole year for each) representing distinct phases of dental development: (1) children aged 1–5 years representing the primary dentition; (2) children aged 6–11 years representing the mixed dentition; (3) youths aged 12–17 years representing the permanent dentition during adolescence; and (4) adults aged 18–59 years representing the permanent dentition during adulthood. Reflecting the recruitment strategy, most adults were of child-rearing age in their 20 s to 40 s. Therefore we did not attempt to further partition the adult samples into finer age strata. Edentulous participants were excluded from the present analyses. This analysis plan required breaking the family structure of the sample.

Because dental caries indices exhibit skewed distributions, we used the nonparametric Mann-Whitney *U* test to determine sex differences in each age stratum. Furthermore, we modeled the effect of sex on caries indices while adjusting for age using linear regression. We also used linear regression to model caries indices in the total COHRA1 sample with sex, age, and state of residence as predictors in order to assess differences between WV and PA samples. Differences in the age distributions and means between WV and PA samples were tested using the Kolmogorov-Smirnov (K-S) and *t*-tests. Some nonindependence due to biological relationships remained even after partitioning the COHRA1 cohort into site-, age-, and sex-stratified subsamples. We have previously employed two strategies to explore the impact of this residual covariance structure in the data: (1) a mixed-models (i.e., variance components) approach that incorporates the family relationships into the model [16] and (2) performing analysis in the maximum set of unrelated individuals [17, 18]. Both methods showed that results were unchanged. Therefore, for the present study, we used straightforward nonparametric analyses as the primary method for inference, while cautioning that exact *p* values may be inflated. All analyses were performed using the R statistical environment (R Foundation for Statistical Computing, Vienna, AU).

## 3. Results

The breakdown of participants from the COHRA1 sample is shown in Table 1. All age-stratified groups were well-represented in the COHRA1 sample, for both WV and PA, except for individuals over 60 years of age, who were excluded from analyses due to insufficient sample size. WV participants were nearly all white, with 2% cumulatively reporting as black, Hispanic, “Other,” or “No response.” The majority (67%) of the PA sample was also non-Hispanic white, with 25% black, 1% Hispanic, 4% “Other,” and 3% “No response.” There were approximately equal numbers of female adults and female children, whereas there were substantially fewer male adults than male children, for both WV and PA.

**Figure 1:**
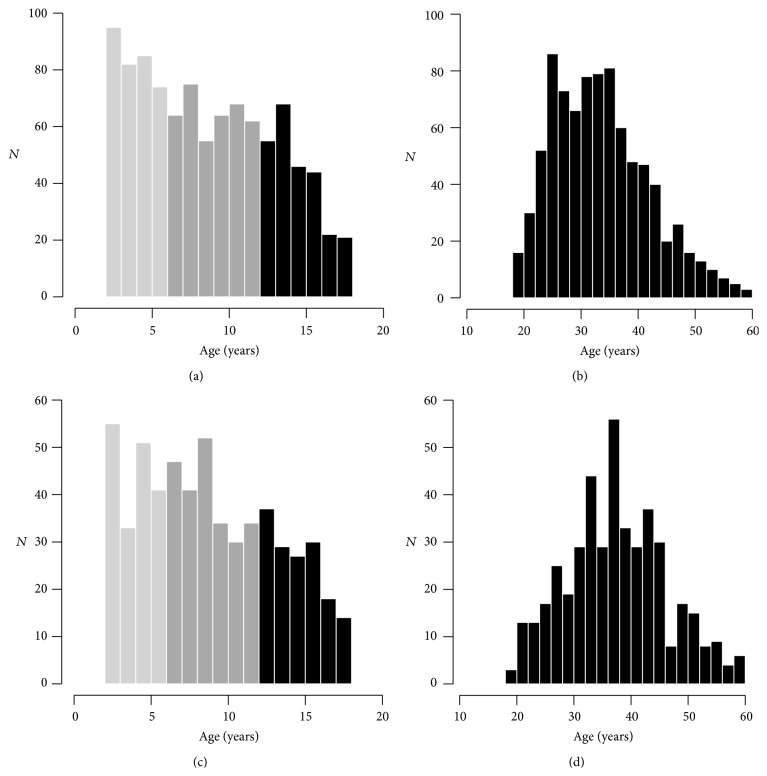
Histograms showing age distributions of the COHRA1 sample. (a) WV children, (b) WV adults, (c) PA children, and (d) PA adults. Light gray, dark gray, and black bars represent, respectively, the participants for whom primary, mixed, and permanent dentition was considered in this study. Age distributions were similar between WV and PA children, whereas WV adults were significantly younger than PA adults.

Four age strata were considered which represent different phases of dental development: primary dentition (ages 1–5), mixed dentition (ages 6–11), adolescent permanent dentition (ages 12–17), and adult permanent dentition (ages 18–59). The distributions of ages in each of these phases are shown in Figure 1. For all childhood strata (Figures 1(a) and 1(c)) age distributions and means were similar between WV and PA (*p* values > 0.05 for all). On the other hand, the age distribution and means differed between WV and PA adults (Figures 1(b) and 1(d); K-S and *t*-test *p* values < 10^−12^), reflecting the comparatively younger WV adult sample. Among adults from 18 to 59 years of age, 4.1% (3.7% of women and 5.0% of men) were edentulous in the PA sample, and 5.3% (4.9% of women and 5.9% of men) were edentulous in the WV sample. Rates of edentulism were not statistically different between women and men nor between PA and WV samples. Edentulous participants were excluded from the following analyses of sex disparities in dental caries.

**Figure 2:**
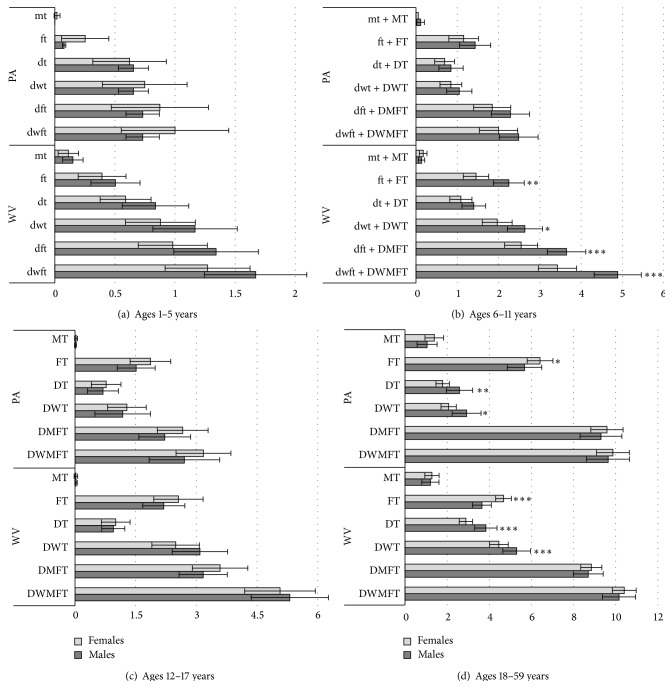
Mean dental caries indices by sex for (a) primary dentition in children aged 1–5 years, (b) mixed dentition in children aged 6–11 years, (c) permanent dentition in adolescents aged 12–17 years, and (d) permanent dentition in adults aged 18–59 years. Error bars represent 95% confidence intervals around the unadjusted means. Significant sex differences are indicated as follows: ^*∗*^ *p* value < 0.05; ^*∗∗*^ *p* value < 0.01; ^*∗∗∗*^ *p* value < 0.001. Note: sex differences were tested while simultaneously adjusting for age; therefore, significant *p* values may be indicated for some caries indices where 95% confidence intervals appear to overlap.

Figure 2 shows the unadjusted means of caries indices in males and females for each age strata in WV and PA separately. Though not statistically significant, children in the PA sample aged 1–5 years exhibited the usual trend with girls experiencing greater dental caries experience in the primary dentition. Conversely, in the WV sample the opposite (nonsignificant) trend was seen, with girls aged 1–5 having lower scores for all caries indices. In ages 6–11, this protective trend was significant in WV, with girls having significantly fewer dental restorations, fewer white spots or decayed teeth, and lower dft + DMFT scores in the mixed dentition than did boys. We further explored this result by separating the mixed dentition into primary and permanent teeth; both dentitions exhibited this protective trend in WV girls aged 6–11, although tests were statistically significant in the primary dentition only. That WV girls had comparatively better dental caries indices than boys is an intriguing result and runs opposite to national [19–22] and international [23–28] trends.

By ages 12–17, the sex disparities in caries experience were not observed, with similar scores for WV girls and boys for each of the caries indices. Likewise, in PA adolescents aged 12–17, girls and boys showed similar caries indices, and the usual (nonsignificant) trend was seen, with girls having greater scores for all indices. In adults, both WV and PA samples showed that women had significantly more dental restorations, whereas men had more teeth with frank decay. DMFT indices, which include evidence of caries from both restored and currently decayed teeth, were not statistically different between the sexes, although the (nonsignificant) trends in both WV and PA were for women to have greater DMFT scores. Note that nonparametric and age-adjusted tests yielded similar results for WV. However, the sex differences observed for caries indices in the PA group aged 18–59 years were only significant with the age-adjustment.

Though not the focus of this study, significantly poorer scores in WV compared to PA were observed for virtually every caries index in all age strata (results not shown), with a few notable exceptions such as DMFT and DWMFT in the adults and MT in children aged 6–11 and 12–17 years, which were not different between WV and PA.

Sex differences were also tested within each PA recruitment site, which followed the trends observed in the total PA sample (results not shown). However, significant sex differences were observed for children aged 1–5 (with girls exhibiting greater caries scores) in both Bradford (*p* values < 0.01 for dwft, dft, dt, and dwt) and Braddock (*p* values < 0.05 for dwft and dwt) subsamples. These results are consistent with the (nonsignificant) trends observed in the total PA sample.

## 4. Discussion

This study addressed the question of whether sex differences in dental caries experience were observed across phases of dental development in two Northern Appalachian populations. Four key insights into dental caries experience in these Appalachian populations were observed: (1) in the WV sample, girls experienced greater protection against caries compared to boys, with the trend readily apparent in children aged 1–5 and significant in those aged 6–11; (2) by ages 12–17 WV girls matched boys with respect to caries; (3) in adults from both WV and PA populations, women had undergone more dental restorations, whereas men had more current decay; and (4) the WV sample, across all ages, exhibited a greater burden of dental caries than did PA according to nearly all caries indices.

Especially intriguing was the evidence that young WV girls experienced protection compared to boys, which counters the trend usually seen for sex differences in dental caries. Anecdotal evidence from oral health professionals in WV indicates that greater cultural importance is placed on girls' dental aesthetics, which is consistent with the significantly lower caries indices observed in WV girls aged 6–11 years as well as the trend observed in younger girls aged 1–5 years. However, during adolescence girls caught up with boys and, by adulthood, surpassed men for some indices. Similar changing trends across age/phases of dental development were not observed in PA. It is unknown what differences accounted for the early protection seen in WV girls, or equivalently what factors contributed to the increased risk seen in WV boys.

In adults, the sex differences were not apparent in the total number of teeth affected (e.g., DMFT) but instead in the indices representing restorations and current decay. Therefore, these sex differences could be masked in studies using only DMFT to measure disease, which is indeed a common approach in epidemiological studies of caries. Moreover, the fact that women exhibited fewer teeth with current decay and more dental restorations suggests that women may have utilized dental health care to a greater degree than did men. This, combined with the greater burden of dental caries experienced by young WV boys, indicates that in some Appalachian populations, males, rather than females, may be the disadvantaged sex.

The scope of this study was simply to assess potential sex differences in dental caries experience. We did not attempt to determine the causes of these differences. Therefore, additional work is needed to identify the factors contributing to these sex disparities and, ultimately, to develop strategies to reduce them. Likewise, we did not specifically address the roles of race or socioeconomic factors, which certainly impact risk of dental caries, although we do not expect that demography fully accounts for the observed sex differences. Race distributions were balanced between the sexes, and very few non-whites were present in WV sample, though substantial racial and ethnic minorities were included in the PA sample. Race may have contributed to differences between WV and PA (although differences were observed even if we limited analysis to whites only; results are not shown) but likely not sex differences within each population. Indices of socioeconomic status are typically defined at the household level, usually measured as the combined incomes of both male and female household members. Single-parent households, which may be more often headed by women, may be economically disadvantaged compared to two-parent households. This could partly explain sex differences seen in adults, but not children (as child's sex is not correlated with family structure).

Our primary statistical approach for this study was to compare males and females within site and age strata using nonparametric methods. This approach was robust to the zero-inflated and skewed distributions of the caries indices; however, it did not specifically incorporate the covariance structure due to relative pairs into the analysis. On average, the presence of some male-male and female-female relative pairs within an age stratum could inflate the sex differences, whereas the presence of male-female relative pairs within an age stratum could deflate the sex differences. In theory, the number of same-sex (i.e., male-male plus female-female) relative pairs is equal to the number of male-female relative pairs, and therefore the opposing effects would partly counteract each other. Possible assortative mating (i.e., clustering of cohabiting adult spouses/mates with respect to oral health) would bias the analyses toward the null hypothesis of no sex differences. Therefore, it is unlikely that our statistical approach could lead to false positive detection of sex differences.

A limitation of this study is that we have extrapolated the life course from cross-sectional data, whereas the observed trends may in truth represent differences in WV birth cohorts rather than changes in sex disparities with age. Nevertheless, this alternative interpretation (i.e., differences in disparities between birth cohorts) is equally interesting and leads to a similar set of questions that need to be answered before sex disparities can be resolved. Also, this study did not address dental caries in the elderly, which is a vulnerable population that experiences decline in oral health with advanced age. These two limitations did not impact our detection of sex differences; however, they may impact our interpretation of the apparent changes in sex differences across the lifespan.

Other important limitations of this study are volunteer bias and the lack of generalizability to other communities in Appalachia. Though COHRA1 participants are similar in many important dimensions (income, ages, educational attainment, etc.) to the underlying communities in the catchment area, we are unable to address whether our sample differs with respect to indices of oral health. Furthermore, the observed sex differences could be confounded if the relationship between volunteerism and oral health differs by sex. For example, the observed differences could be overstated if healthy (or unhealthy) individuals of one sex were more likely to participate. Moreover, because Appalachia comprises many heterogeneous populations and our recruitment strategy did not target some groups (such as those living alone, without offspring, or with adult-aged offspring) our results are not generalizable to other populations.

## 5. Conclusions

We provide evidence that, compared to males, Northern Appalachian females in WV experience protection against dental caries during childhood but not during adolescence or adulthood. Furthermore, in both WV and PA, adult women had more dental restorations, whereas men had more current decay, suggesting a sex bias in accessing or utilizing dental health services. These results indicate oral health disparities are present in these Northern Appalachian populations; more efforts are needed to identify the causes of such disparities and to formulate strategies to reduce them.

## Figures and Tables

**Table 1:** Composition of the COHRA1 sample as indicated by sample size for each stratum.

Sample size	All		WV		PA		PA subsamples					
							BU		BR		BK	
	M	F	M	F	M	F	M	F	M	F	M	F
Age group												
Ages 1–5	358	334	252	221	106	113	34	37	43	45	29	31
Ages 6–11	324	302	197	191	127	111	40	45	37	27	50	39
Ages 12–17	201	209	120	135	81	74	32	21	16	16	33	37
Ages 18–59	482	845	319	552	163	293	68	99	60	97	35	97
Ages >60	5	7	4	6	1	1	1	0	0	0	0	1
Race or ethnicity												
White	1193	1470	864	1084	329	386	144	168	145	170	40	48
Black	118	159	8	4	110	155	20	24	2	1	88	130
Hispanic	9	15	6	6	3	9	0	2	2	4	1	3
Other	26	32	8	3	18	29	2	1	4	4	12	24
No response	24	21	6	8	0	0	9	7	3	6	6	0
Total	1370	1697	892	1105	478	592	175	202	156	185	147	205

BU = Burgettestown; BR = Bradford; BK = Braddock.
